# Fibroblast growth factor 23 as a risk factor for incident diabetes

**DOI:** 10.1097/MNH.0000000000001078

**Published:** 2025-04-15

**Authors:** Martin H. de Borst

**Affiliations:** Department of Internal Medicine, Division of Nephrology, University Medical Center Groningen, University of Groningen, Groningen, The Netherlands

**Keywords:** cardiovascular-kidney-metabolic syndrome, diabetes, insulin resistance, mineral metabolism

## Abstract

**Purpose of review:**

Diabetes is a major global health concern, affecting millions and increasing morbidity and mortality. Recent research highlights fibroblast growth factor 23 (FGF23) as a potential contributor to type 2 diabetes and its cardiovascular complications. This review explores the role of FGF23 in metabolic and cardiovascular dysfunction and discusses possible therapeutic interventions.

**Recent findings:**

Deregulated FGF23 is linked to insulin resistance, pancreatic β-cell dysfunction, and systemic inflammation. Studies suggest FGF23 influences glucose metabolism via insulin signaling, oxidative stress, and inflammation. Epidemiological data indicate that elevated FGF23 levels are associated with an increased risk of type 2 diabetes and posttransplant diabetes, independent of traditional risk factors. Higher FGF23 levels have also been linked with an increased cardiovascular risk in patients with diabetes, even without chronic kidney disease.

**Summary:**

FGF23 is emerging as a key factor in the cardiovascular-kidney-metabolic syndrome, connecting diabetes and cardiovascular disease. While studies suggest consistent associations, causal mechanisms remain unclear. No therapies specifically target FGF23 to lower diabetes risk, but fibroblast growth factor receptor 4 (FGFR4) inhibitors show promise. Future research should examine the role of FGF23 in individuals with normal kidney function and explore whether modifying its levels could reduce diabetes and cardiovascular risk.

## INTRODUCTION

Diabetes is among the most common noncommunicable diseases, affecting one in ten individuals or 537 million adults worldwide in 2021 [1]. This figure is expected to rise to 643 million by 2030 and to 783 million by 2045. Diabetes can lead to microvascular (retinopathy, neuropathy, nephropathy) and macrovascular (peripheral vascular disease, stroke, and coronary artery disease) complications, driving a three-fold increased risk of developing cardiovascular disease, compared to individuals without diabetes [2,3]. Given the major impact of diabetes and its complications on patient outcomes and healthcare resources, diabetes prevention strategies are a major focus of research.

Risk factors for the development of type 2 diabetes, the most common form of diabetes, include overweight/obesity, age over 45, a positive family history, physical inactivity and ethnicity. The presence of metabolic dysfunction-associated fatty liver disease (MAFLD) also predisposes to the development of type 2 diabetes. In patients who already have type 2 diabetes, the risk of cardiovascular events is driven by traditional risk factors such as dyslipidemia, smoking, and hypertension, as well as other factors including diabetic nephropathy. Calcification of the vascular media wall is one of the hallmarks of advanced diabetes, and the presence of extensive vascular calcifications is an independent predictor of adverse cardiovascular outcomes, both in individuals with and without diabetes [4]. While the pathophysiology of media calcification in diabetes has not yet been fully elucidated, several mechanisms seem to play a role. First, media calcification is considered a process of hydroxyapatite mineralization within the medial layer. The loss of endogenous mineralization inhibitors such as matrix gla protein and pyrophosphate has been associated with vascular stiffness and calcification in type 2 diabetes [5,6]. Vascular smooth muscle cells can undergo a phenotypic change towards osteoblast-like cells that express and release osteochondric proteins [7]. Lastly, elevated plasma calcium or phosphorus levels promote apatite nucleation and crystal growth, further promoting vascular calcification.

It has been known since the 1980s that patients with type 2 diabetes have a lower bone mass, compared to individuals without diabetes [8,9]. Some studies suggested that these bone abnormalities, that also contribute to an increased fracture risk in type 2 diabetes, are related to co-existing microvascular complications [10]. At the same time, several studies point towards discrete yet detectable changes in mineral metabolism that already start to occur in early diabetes. These changes include lower levels of parathyroid hormone and higher levels of the phosphaturic hormone fibroblast growth factor 23 (FGF23) [11]. Of note, more recent studies also point towards a potential role for FGF23 as a risk factor, or potentially even a contributor to, the development of diabetes. Thus, a potential dual role for FGF23 (deregulation) has been proposed in both the development of type 2 diabetes and its complications.

Therefore, the aim of this review is to provide an overview of the present literature on FGF23 as a risk factor for type 2 diabetes, as well as the implications of deregulated FGF23 for cardiovascular risk in patients with diabetes. 

**Box 1:**
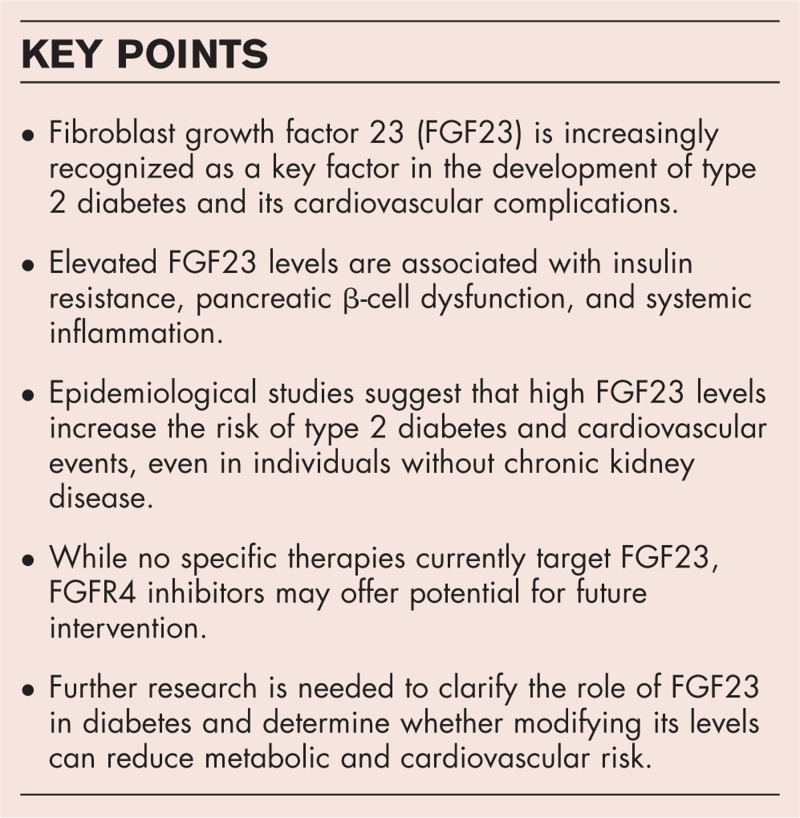
no caption available

## PHYSIOLOGY OF FIBROBLAST GROWTH FACTOR 23

Long before the discovery of the phosphaturic hormone FGF23 it was recognized that a phosphatonin must exist to regulate phosphate excretion. In the year 2000, mutations in the *FGF23* gene that render the protein resistant to proteolytic cleavage were identified as the cause of the rare disease autosomal dominant hypophosphatemic rickets (ADHR) [12]. FGF23 is a circulating hormone that participates in the regulation of phosphate by decreasing phosphate reabsorption in the proximal tubuli of the kidney, and in the regulation of vitamin D metabolism by suppressing expression of the enzyme 1-alpha hydroxylase [13,14]. Subsequently, excessive FGF23 levels were also discovered to be a causative factor in X-linked hypophosphatemic rickets and tumor-induced osteomalacia [15,16].

FGF23 is predominantly expressed and secreted by osteocytes in bone. FGF23 signaling can take place through several routes: a canonical route where FGF23 binds to the FGF receptor 1c (FGFR1c) at the proximal tubule in the presence of the co-factor Klotho, inducing phosphaturia and suppressing vitamin D activation; a klotho-independent route through another FGF receptor (FGFR4), activating distinct intracellular pathways (calcineurin/NFAT), promoting left ventricular hypertrophy in the heart, and through a noncanonical route involving a circulating, soluble form of Klotho that can also bind to FGF23 receptors [17^▪^]. Finally, Klotho can convert the FGFR1(IIIc) receptor into a specific FGF23 receptor [18]. FGF23 can be cleaved into C-terminal fragments that may serve specific roles, such as increasing the bioavailability of iron during inflammation [19^▪▪^].

Patients with impaired kidney function have higher circulating FGF23 levels, compared to those with normal kidney function [20]. Moreover, higher levels of FGF23 have been strongly linked with an increased mortality risk in hemodialysis patients [21]; a finding that was later replicated in various chronic kidney disease (CKD) and non-CKD populations. FGF23 levels increase relatively early during CKD, before other components of mineral metabolism such as PTH, phosphate start to rise.

In addition to the effects of FGF23 on phosphate and vitamin D metabolism, additional effects (so-called “off-target effects”) have been described. Animal studies have shown that FGF23 can induce left ventricular hypertrophy (LVH) through FGFR4 [22,23], and in patients higher FGF23 levels predispose to development of LVH [24–26]. Further, FGF23 has been linked with impaired glucose metabolism. This link has been established by the observation that Fgf23-knockout mice are hypoglycemic and have profoundly increased peripheral insulin sensitivity and improved subcutaneous glucose tolerance [27]. These abnormalities were absent in Fgf23/vitamin D receptor double-knockout mice, indicating that vitamin D mediates the relationship between FGF23 and glucose metabolism [27]. Moreover, HYP-mice with a PHEX (phosphate regulating gene with homologies to endopeptidases located on the X chromosome) mutation that leads to FGF23 overexpression also display hyperglycemia and hypoinsulinemia [28]. Klotho mutant (kl/kl) mice display decreased pancreatic insulin content and increased insulin sensitivity, compared to heterozygous and wild-type mice [29]. In contrast, mice with Klotho overexpression have higher blood insulin levels, require less glucose infusion to maintain blood glucose levels, and show significantly less pronounced hypoglycemic responses to insulin and insulin-like growth factor-1, compared to wild-type mice [30].

## FIBROBLAST GROWTH FACTOR 23 AND (DEREGULATED) GLUCOSE HOMEOSTASIS IN HUMANS

**FIGURE 1:**
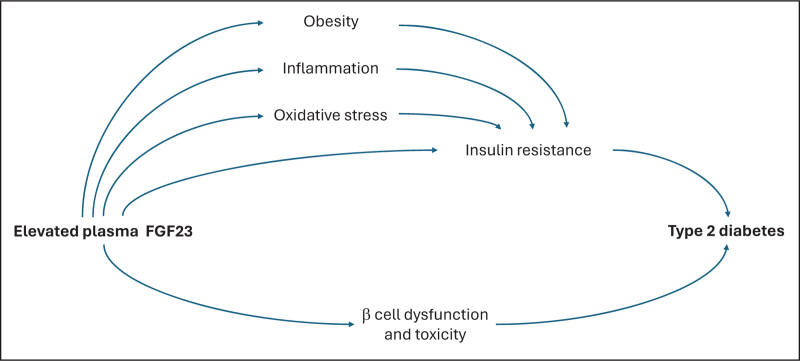
Hypothesized mechanisms of FGF23-induced incident diabetes. Overview of proposed mechanisms by which a higher plasma FGF23 level could lead to an increased risk of type 2 diabetes. Details are provided in the text. FGF23, fibroblast growth factor 23.

Deregulated FGF23 and klotho are involved in impaired insulin signaling and insulin resistance through various potential mechanisms (Fig. 1). In the setting of insulin resistance, insulin signaling is impaired at the level of tyrosine phosphorylation of insulin receptor substrate (IRS)-1, which leads to downregulation of the phosphatidylinositol 3 kinase (PI3K)/AKT pathway and subsequent muscle wasting. Klotho seems to interfere with insulin receptor signaling and downstream processes, contributing to muscle wasting and insulin resistance [31^▪^,32^▪^,^33^]. Furthermore, higher plasma FGF23 levels have been linked with a higher BMI and insulin resistance in both healthy individuals and patients with diabetes (Table 1).

**Table 1:** Cross-sectional studies linking FGF23 with parameters of glucose metabolism and insulin resistance

Population	Parameter(s) associated with FGF23	Reference
Total FGF23 (C-terminal and intact FGF23)		
General population	BMI, fat mass, insulin sensitivity, HOMA-IR, postload OGTT	[51]
General population	Glucose, insulin, proinsulin	[52^▪▪^]
Stage 3–5 CKD	Insulin resistance	[55]
Intact FGF23		
General population	BMI, insulin sensitivity	[51]
Obese adolescents	Insulin resistance, HOMA-IR	[65]
Type 2 diabetes	Serum resistin levels	[66]
Stage 3–5 CKD	HOMA-IR	[54]

BMI, body mass index; CKD, chronic kidney disease; FGF23, fibroblast growth factor 23; HOMA-IR, homeostasis model assessment of insulin resistance; OGTT, oral glucose tolerance test.

Another route by which FGF23 may contribute to the risk of diabetes is via direct and indirect effects on pancreatic β-cell function. One such effect is through ferroptosis, a form of nonapoptotic regulated cell death accompanied by iron-dependent lipid peroxide accumulation. Ferroptosis is involved in impaired glucose-induced insulin secretion [34,35], and FGF C-terminal fragments promote ferroptosis [19^▪▪^]. These results pointing towards a potentially deleterious effect of FGF23 on pancreatic β-cells were recently balanced by another study suggesting that FGF23 partially protects β-cells against glucolipotoxicity through interactions with the stimulus-secretion cascade [36^▪▪^]. The same study also demonstrated that FGF23 impairs glucose-induced insulin release in murine islets [36^▪▪^]. Thus, while these findings do not exclude the possibility that FGF23 plays a role in the development of diabetes through direct toxic effects on the pancreas, the other side of the coin could be that FGF23 provides β-cell protection in established diabetes, during high-glucose and high-lipid conditions.

In addition, inflammation is an important trigger of both FGF23 production and new-onset diabetes, and as such a pro-inflammatory environment could (partly) explain higher FGF23 levels in individuals prone to develop diabetes [37]. Several studies have shown associations between FGF23 and markers of inflammation [38–41]. In CKD, FGF23 can directly stimulate hepatic secretion of inflammatory cytokines, potentially contributing to the pro-inflammatory milieu [42^▪^]. FGF23 has also been shown to contribute to inflammation in other organ systems and diseases, including pulmonary diseases such as cystic fibrosis [43].

Furthermore, FGF23 induces oxidative stress and cellular senescence in human mesenchymal stem cells from skeletal muscle, a mechanism potentially contributing to insulin resistance [31^▪^,^44^]. The FGF23 co-receptor klotho facilitates reactive oxygen species removal and confers oxidative stress resistance, potentially contributing to the antiaging properties of klotho [45]. Since both chronic kidney disease and oxidative stress itself can reduce klotho [46], a vicious cycle characterized by low klotho and increased oxidative stress could promote the risk of diabetes. Finally, FGF23 may not be a cause of incident diabetes, but rather an expression of deregulated mineral metabolism in early diabetes.

Patients with both type 1 and type 2 diabetes are more likely to display deregulations in bone and mineral metabolism including elevated FGF23 levels [47–49], compared to individuals without diabetes [11,50]. Several cross-sectional studies have shown that circulating FGF23 levels are associated with parameters of glucose homeostasis as well as insulin sensitivity/resistance (Table 1). These associations appear to be mediated by BMI [51,52^▪▪^], and weight loss is accompanied by a decrease in intact FGF23 levels [51]. In one study, FGF23 was positively associated with HOMA-IR, BMI and waist circumference in individuals without CKD, but not among individuals with CKD [53]. This may be explained by impaired kidney function, boosting FGF23 levels to very high levels and overriding potential effects related to glucose metabolism. In contrast, other studies did report associations between plasma FGF23 and insulin resistance in CKD patients [54,55]. Serum klotho levels have also been independently associated with HOMA-IR in patients with type 2 diabetes and stage 2–3 CKD [56].

While cross-sectional studies reviewed above strongly suggest a relationship between FGF23 levels and (deregulated) glucose homeostasis, they do not clarify whether the development of insulin resistance or diabetes drives abnormal mineral homeostasis, the opposite, or both. It is long known that insulin promotes a shift of phosphate into the intracellular compartment [57]. Insulin, contrary to FGF23, directly stimulates the NaPi-II co-transporter, promoting renal phosphate reabsorption at the proximal tubuli of the kidney [58]. Furthermore, multiple human studies have reported changes in FGF23 and phosphate in response to glucose loading (Table 2). Ursem *et al.* reported that an oral glucose load in vitamin D-deficient patients with impaired glucose metabolism led to a decrease plasma (total) FGF23, which could not be attributed to changes in insulin concentration as a euglycemic-hyperinsulinemic clamp did not affect FGF23 levels [59^▪^]. In line, Van der Vaart *et al.* reported that oral glucose loading in nondiabetic individuals was followed by a decrease and subsequent recovery in total FGF23 levels that preceded changes in plasma phosphate levels [52^▪▪^]. In another study in patients with type 2 diabetes, a euglycemic-hyperinsulinemic clamp led to an increase in serum FGF23 levels [60]. Together, these studies suggest that insulin and glucose have opposed effects on FGF23 levels, but these effects may also be context-dependent, as insulin clamping did not lead to increased FGF23 levels in individuals without diabetes [60]. At the same time, epidemiological studies point towards a role for FGF23 in the development of diabetes.

**Table 2:** Reported effects of insulin and glucose on FGF23 levels

		Effect on			
Intervention	Population	Glucose	Insulin	FGF23	Ref
Oral glucose loading (75 g)	Nondiabetes	↑	↑	↓	[52^▪▪^]
Oral glucose loading (75 g)	Prediabetes	↑	↑	↓	[59^▪^]
Euglycemic-hyperinsulinemic clamp	Nondiabetes	=	↑	=	[60]
Euglycemic-hyperinsulinemic clamp	Prediabetes	=	↑	=	[59^▪^]
Euglycemic-hyperinsulinemic clamp	Type 2 diabetes	=	↑	↑	[60]

FGF23, fibroblast growth factor 23.

## EPIDEMIOLOGICAL EVIDENCE CONNECTING FIBROBLAST GROWTH FACTOR 23 WITH DIABETES RISK

In line with the preceding lines of evidence that connect higher FGF23 levels with deregulated glucose metabolism and insulin resistance, several studies have demonstrated that individuals without diabetes who have a higher FGF23 level are at increased risk of developing type 2 diabetes. In a population-based cohort of 5482 participants, we found that a higher baseline C-terminal FGF23 level was associated with the development of diabetes, independent of age, sex, plasma calcium parathyroid hormone, 25(OH) vitamin D, phosphate, proinsulin and HDL cholesterol, smoking, systolic blood pressure, alcohol use and kidney function [52^▪▪^]. Interestingly, the association between FGF23 and incident diabetes lost significance after additional adjustment for BMI and FGF23 itself was also associated with the development of obesity. Subsequently, we also found that a higher FGF23 level was associated with a higher risk of developing posttransplant diabetes in a cohort of kidney transplant recipients [61^▪^]. A higher plasma FGF23 level also represents a risk factor for an increased risk of premature mortality and major adverse cardiovascular events in individuals with type 2 diabetes [62^▪▪^]. Interestingly, this association was also present in individuals with diabetes and normal or mildly impaired kidney function (eGFR > 60 ml/min/1.73 m^2^).

## CONCLUSION

The cardiovascular-kidney-metabolic syndrome, defined as a health disorder that is attributable to connections among obesity, diabetes, CKD, and cardiovascular disease, is gaining increasing attention. This review summarized recent evidence that positions the phosphaturic hormone FGF23 as a potentially important factor in the development of diabetes as well as its cardiovascular complications. Of interest, while most studies so far focused on populations with CKD, these relationships also seem to be present in individuals with normal kidney function. These initial observations should stimulate further research addressing the relationships of deregulated FGF23 and other factors involved in mineral metabolism with the risks of developing diabetes and cardiovascular disease in populations with normal kidney function.

Another question is whether lowering of FGF23 might reduce the risk of developing diabetes. Unfortunately, no specific therapies are available to reduce plasma FGF23, block its receptors or increase levels of klotho in patients. While a high-calcium and phosphate diet-induced increase in plasma FGF23 did not influence fasting insulin and glucose concentrations [59^▪^], the nonspecific nature of the intervention or short follow-up (3 days) may have blunted potential effects of FGF23. Of interest, FGFR4 inhibitors are currently being developed and tested in cancer, opening potential for downstream targeting of high FGF23 levels in CKD patients to prevent the development of left ventricular hypertrophy and heart failure [63,64^▪▪^].

Overall, FGF23 has a well documented role as a regulator of mineral metabolism as well as a cardiovascular risk factor in CKD. Beyond that, emerging data suggest that deregulated FGF23 contributes to the cardiovascular-kidney-metabolic syndrome through various mechanisms, including the risk of developing diabetes. The exact role of FGF23 in individuals with normal kidney function remains to be addressed in future studies, paving the way for intervention studies as more specific therapies become available.

## Acknowledgements

*None.*

### Financial support and sponsorship

*None.*

### Conflicts of interest

*There are no conflicts of interest.*

## REFERENCES AND RECOMMENDED READING

Papers of particular interest, published within the annual period of review, have been highlighted as:

▪ of special interest
▪▪ of outstanding interest

## References

[1] International Diabetes Federation. IDF diabetes atlas. 10th ed. Brussels, Belgium; 2021. Available at: https://www.diabetesatlas.org/.

[2] Kannel WB McGee DL. Diabetes and cardiovascular disease: the Framingham study. JAMA 1979; 241:2035–2038. 430798 10.1001/jama.241.19.2035

[3] Tancredi M Rosengren A Svensson AM. Excess mortality among persons with type 2 diabetes. N Engl J Med 2015; 373:1720–1732. 26510021 10.1056/NEJMoa1504347

[4] Raggi P Shaw LJ Berman DS Callister TQ. Prognostic value of coronary artery calcium screening in subjects with and without diabetes. J Am Coll Cardiol 2004; 43:1663–1669. 15120828 10.1016/j.jacc.2003.09.068

[5] Sardana M Vasim I Varakantam S. Inactive matrix Gla-protein and arterial stiffness in type 2 diabetes mellitus. Am J Hypertens 2017; 30:196–201. 27927630 10.1093/ajh/hpw146

[6] Liabeuf S Olivier B Vemeer C. Vascular calcification in patients with type 2 diabetes: the involvement of matrix Gla protein. Cardiovasc Diabetol 2014; 13:9. 24762216 10.1186/1475-2840-13-85 PMC4017083

[7] Tyson KL Reynolds JL McNair R. Osteo/chondrocytic transcription factors and their target genes exhibit distinct patterns of expression in human arterial calcification. Arterioscler Thromb Vasc Biol 2003; 23:489–494. 12615658 10.1161/01.ATV.0000059406.92165.31

[8] Vincenti F Arnaud SB Recker R. Parathyroid and bone response of the diabetic patient to uremia. Kidney Int 1984; 25:677–682. 6482171 10.1038/ki.1984.73

[9] Andress DL Hercz G Kopp JB. Bone histomorphometry of renal osteodystrophy in diabetic patients. J Bone Mineral Res 1987; 2:525–531. 10.1002/jbmr.5650020609 3455634

[10] Shanbhogue VV Hansen S Frost M. Bone disease in diabetes: another manifestation of microvascular disease? Lancet Diabetes Endocrinol 2017; 5:827–838. 28546096 10.1016/S2213-8587(17)30134-1

[11] Yeung SMH Bakker SJL Laverman GD De Borst MH. Fibroblast growth factor 23 and adverse clinical outcomes in type 2 diabetes: a bitter-sweet symphony. Curr Diab Rep 2020; 20:50. 32857288 10.1007/s11892-020-01335-7 PMC7455586

[12] ADHR Consortium. Autosomal dominant hypophosphataemic rickets is associated with mutations in FGF23. Nat Genet 2000; 26:345–348. 11062477 10.1038/81664

[13] Saito H Kusano K Kinosaki M. Human fibroblast growth factor-23 mutants suppress Na+-dependent phosphate co-transport activity and 1alpha,25-dihydroxyvitamin D3 production. J Biol Chem 2003; 278:2206–2211. 12419819 10.1074/jbc.M207872200

[14] Shimada T Hasegawa H Yamazaki Y. FGF-23 is a potent regulator of vitamin D metabolism and phosphate homeostasis. J Bone Miner Res 2004; 19:429–435. 15040831 10.1359/JBMR.0301264

[15] Shimada T Mizutani S Muto T. Cloning and characterization of FGF23 as a causative factor of tumor-induced osteomalacia. Proc Natl Acad Sci USA 2001; 98:6500–6505. 11344269 10.1073/pnas.101545198 PMC33497

[16] Jonsson KB Zahradnik R Larsson T. Fibroblast growth factor 23 in oncogenic osteomalacia and X-linked hypophosphatemia. N Engl J Med 2003; 348:1656–1663. 12711740 10.1056/NEJMoa020881

[17▪] Edmonston D Grabner A Wolf M. Excellent review summarizing the literature on FGF23 and klotho in cardiovascular disease. FGF23 and klotho at the intersection of kidney and cardiovascular disease. Nat Rev Cardiol 2024; 21:11–24. 37443358 10.1038/s41569-023-00903-0

[18] Urakawa I Yamazaki Y Shimada T. Klotho converts canonical FGF receptor into a specific receptor for FGF23. Nature 2006; 444:770–774. 17086194 10.1038/nature05315

[19▪▪] Courbon G Thomas JJ Martinez-Calle M. Bone-derived C-terminal FGF23 cleaved peptides increase iron availability in acute inflammation. Blood 2023; 142:106–118. 37053547 10.1182/blood.2022018475 PMC10356820

[20] Larsson T Nisbeth U Ljunggren O. Circulating concentration of FGF-23 increases as renal function declines in patients with chronic kidney disease, but does not change in response to variation in phosphate intake in healthy volunteers. Kidney Int 2003; 64:2272–2279. 14633152 10.1046/j.1523-1755.2003.00328.x

[21] Gutiérrez OM Mannstadt M Isakova T. Fibroblast growth factor 23 and mortality among patients undergoing hemodialysis. N Engl J Med 2008; 359:584–592. 18687639 10.1056/NEJMoa0706130 PMC2890264

[22] Faul C Amaral AP Oskouei B. FGF23 induces left ventricular hypertrophy. J Clin Invest 2011; 121:4393–4408. 21985788 10.1172/JCI46122 PMC3204831

[23] Grabner A Amaral AP Schramm K. Activation of cardiac fibroblast growth factor receptor 4 causes left ventricular hypertrophy. Cell Metab 2015; 22:1020–1032. 26437603 10.1016/j.cmet.2015.09.002 PMC4670583

[24] Jovanovich A Ix JH Gottdiener J. Fibroblast growth factor 23, left ventricular mass, and left ventricular hypertrophy in community-dwelling older adults. Atherosclerosis 2013; 231:114–119. 24125420 10.1016/j.atherosclerosis.2013.09.002 PMC3840534

[25] Mirza MAI Larsson A Melhus H. Serum intact FGF23 associate with left ventricular mass, hypertrophy and geometry in an elderly population. Atherosclerosis 2009; 207:546–551. 19524924 10.1016/j.atherosclerosis.2009.05.013

[26] Smith K Defilippi C Isakova T. Fibroblast growth factor 23, high-sensitivity cardiac troponin, and left ventricular hypertrophy in CKD. Am J Kidney Dis 2013; 61:67–73. 22883134 10.1053/j.ajkd.2012.06.022 PMC3525738

[27] Hesse M Fröhlich LF Zeitz U. Ablation of vitamin D signaling rescues bone, mineral, and glucose homeostasis in Fgf-23 deficient mice. Matrix Biol 2007; 26:75–84. 17123805 10.1016/j.matbio.2006.10.003

[28] Zelenchuk LV Hedge AM Rowe PSN. PHEX mimetic (SPR4-peptide) corrects and improves HYP and wild type mice energy-metabolism. PLoS One 2014; 9:e97326. 24839967 10.1371/journal.pone.0097326 PMC4026222

[29] Utsugi T Ohno T Ohyama Y. Decreased insulin production and increased insulin sensitivity in the klotho mutant mouse, a novel animal model for human aging. Metabolism 2000; 49:1118–1123. 11016890 10.1053/meta.2000.8606

[30] Kurosu H Yamamoto M Clark JD. Physiology: suppression of aging in mice by the hormone Klotho. Science 2005; 309:1829–1833. 16123266 10.1126/science.1112766 PMC2536606

[31▪] Elsurer Afsar R Afsar B Ikizler TA. Fibroblast growth factor 23 and muscle wasting: a metabolic point of view. Kidney Int Rep 2023; 8:1301–1314. 37441473 10.1016/j.ekir.2023.04.027 PMC10334408

[32▪] Unger RH. Klotho-induced insulin resistance: a blessing in disguise? Nat Med 2006; 12:56–57. 16397569 10.1038/nm0106-56

[33] Gu H Jiang W You N. Soluble Klotho improves hepatic glucose and lipid homeostasis in type 2 diabetes. Mol Ther Methods Clin Dev 2020; 18:811–823. 32953932 10.1016/j.omtm.2020.08.002 PMC7479259

[34] Sha W Hu F Xi Y. Mechanism of ferroptosis and its role in type 2 diabetes mellitus. J Diabetes Res 2021; 2021:9999612. 34258295 10.1155/2021/9999612 PMC8257355

[35] Bruni A Pepper AR Pawlick RL. Ferroptosis-inducing agents compromise in vitro human islet viability and function. Cell Death Dis 2018; 9:595. 29789532 10.1038/s41419-018-0506-0 PMC5964226

[36▪▪] Pajaziti B Yosy K Steinberg OV Düfer M. FGF-23 protects cell function and viability in murine pancreatic islets challenged by glucolipotoxicity. Pflugers Arch 2023; 475:309–322. 36437429 10.1007/s00424-022-02772-x PMC9908675

[37] David V Martin A Isakova T. Inflammation and functional iron deficiency regulate fibroblast growth factor 23 production. Kidney Int 2016; 89:135–146. 26535997 10.1038/ki.2015.290 PMC4854810

[38] Mendoza JM Isakova T Ricardo AC. Fibroblast growth factor 23 and inflammation in CKD. Clin J Am Soc Nephrol 2012; 7:1155–1162. 22554719 10.2215/CJN.13281211 PMC3386678

[39] Munoz Mendoza J Isakova T Cai X. Inflammation and elevated levels of fibroblast growth factor 23 are independent risk factors for death in chronic kidney disease. Kidney Int 2017; 91:711–719. 28017325 10.1016/j.kint.2016.10.021 PMC5313324

[40] Hanks L Casazza K. Judd S, one NJP, 2015 undefined. Associations of fibroblast growth factor-23 with markers of inflammation, insulin resistance and obesity in adults. PLoS One 2015; 10:e0122885. 25811862 10.1371/journal.pone.0122885 PMC4374938

[41] Wallquist C Mansouri L Norrbäck M. Associations of fibroblast growth factor 23 with markers of inflammation and leukocyte transmigration in chronic kidney disease. Nephron 2018; 138:287–295. 29301137 10.1159/000485472

[42▪] Singh S Grabner A Yanucil C. Fibroblast growth factor 23 directly targets hepatocytes to promote inflammation in chronic kidney disease. Kidney Int 2016; 90:985–996. 27457912 10.1016/j.kint.2016.05.019 PMC5065745

[43] Krick S Grabner A Baumlin N. Fibroblast growth factor 23 and Klotho contribute to airway inflammation. Eur Resp J 2018; 52:1800236. 10.1183/13993003.00236-2018 PMC6044452 29748308

[44] Sato C Iso Y Mizukami T. Fibroblast growth factor-23 induces cellular senescence in human mesenchymal stem cells from skeletal muscle. Biochem Biophys Res Commun 2016; 470:657–662. 26797283 10.1016/j.bbrc.2016.01.086

[45] Yamamoto M Clark JD Pastor JV. Regulation of oxidative stress by the antiaging hormone klotho. J Biol Chem 2005; 280:38029–38034. 16186101 10.1074/jbc.M509039200 PMC2515369

[46] Christov M Neyra JA Gupta S Leaf DE. Fibroblast growth factor 23 and Klotho in AKI. Semin Nephrol 2019; 39:57–75. 30606408 10.1016/j.semnephrol.2018.10.005

[47] Walsh JS Vilaca T. Obesity type 2 diabetes and bone in adults. Calcif Tissue Int 2017; 100:528–535. 28280846 10.1007/s00223-016-0229-0 PMC5394147

[48] Kasperk C Georgescu C Nawroth P. Diabetes mellitus and bone metabolism. Exp Clin Endocrinol Diab 2017; 125:213–217. 10.1055/s-0042-123036 28073133

[49] Vermeulen S Scheffer-Rath MEA Besouw MTP. Fibroblast growth factor 23 and calcium-phosphate metabolism in relation to cardiovascular risk factors in patients with type 1 diabetes. J Diabetes 2024; 16:e13500. 38124483 10.1111/1753-0407.13500 PMC11128753

[50] Wahl P Xie H Scialla J. Earlier onset and greater severity of disordered mineral metabolism in diabetic patients with chronic kidney disease. Diabetes Care 2012; 35:994–1001. 22446176 10.2337/dc11-2235 PMC3329844

[51] Fernández-Real JM Puig J Serrano M. Iron and obesity status-associated insulin resistance influence circulating fibroblast-growth factor-23 concentrations. PLoS One 2013; 8:e58961. 23555610 10.1371/journal.pone.0058961 PMC3605441

[52▪▪] Van Der Vaart A Eelderink C Van Beek AP. Fibroblast growth factor 23, glucose homeostasis, and incident diabetes: findings of 2 cohort studies. J Clin Endocrinol Metab 2023; 108:971–978. 37139691 10.1210/clinem/dgad246 PMC10505526

[53] Hanks LJ Casazza K Judd SE. Associations of fibroblast growth factor-23 with markers of inflammation, insulin resistance and obesity in adults. PLoS One 2015; 10:e0122885. 25811862 10.1371/journal.pone.0122885 PMC4374938

[54] Fayed A El Nokeety MM Heikal AA. Fibroblast growth factor-23 is a strong predictor of insulin resistance among chronic kidney disease patients. Ren Fail 2018; 40:226–230. 29619868 10.1080/0886022X.2018.1455594 PMC6014287

[55] Garland JS Holden RM Ross R. Insulin resistance is associated with Fibroblast Growth Factor-23 in stage 3-5 chronic kidney disease patients. J Diabetes Complications 2014; 28:61–65. 24125760 10.1016/j.jdiacomp.2013.09.004

[56] Silva AP Mendes F Pereira L. Klotho levels: association with insulin resistance and albumin-to-creatinine ratio in type 2 diabetic patients. Int Urol Nephrol 2017; 49:1809–1814. 28677090 10.1007/s11255-017-1646-3

[57] Kanbay M Vervloet M Cozzolino M. Novel faces of fibroblast growth factor 23 (FGF23): iron deficiency, inflammation, insulin resistance, left ventricular hypertrophy, proteinuria and acute kidney injury. Calcif Tissue Int 2017; 100:217–228. 27826644 10.1007/s00223-016-0206-7

[58] Murer H Hernando N Forster I Biber J. Proximal tubular phosphate reabsorption: molecular mechanisms. Physiol Rev 2000; 80:1373–1409. 11015617 10.1152/physrev.2000.80.4.1373

[59▪] Ursem SR Vervloet MG Büttler RM. The interrelation between FGF23 and glucose metabolism in humans. J Diabetes Complications 2018; 32:845–850. 29996975 10.1016/j.jdiacomp.2018.06.013

[60] Winther K Nybo M Vind B. Acute hyperinsulinemia is followed by increased serum concentrations of fibroblast growth factor 23 in type 2 diabetes patients. Scand J Clin Lab Invest 2012; 72:108–113. 22133206 10.3109/00365513.2011.640407

[61▪] Van Der Vaart A Kremer D Niekolaas T. Time-updated fibroblast growth factor 23 is predictive for posttransplant diabetes mellitus in kidney transplant recipients. J Endocr Soc 2024; 8:bvae055. 38577264 10.1210/jendso/bvae055 PMC10993900

[62▪▪] Yeung SMH Binnenmars SH Gant CM. Fibroblast growth factor 23 and mortality in patients with type 2 diabetes and normal or mildly impaired kidney function. Diabetes Care 2019; 42:2151–2153. 31488569 10.2337/dc19-0528

[63] Chen X Huang Y Chen B. Insight into the design of FGFR4 selective inhibitors in cancer therapy: prospects and challenges. Eur J Med Chem 2024; 263:115947. 37976704 10.1016/j.ejmech.2023.115947

[64▪▪] Fuchs MAA Burke EJ Latic N. Fibroblast growth factor 23 and fibroblast growth factor receptor 4 promote cardiac metabolic remodeling in chronic kidney disease. Kidney Int 2025; S0085-2538:00087-0. 10.1016/j.kint.2025.01.024 PMC12755233 39923962

[65] Wojcik M Janus D Dolezal-Oltarzewska K. The association of FGF23 levels in obese adolescents with insulin sensitivity. J Pediatr Endocrinol Metab 2012; 25 (7–8):687–690. 23155694 10.1515/jpem-2012-0064

[66] Nakashima A Yokoyama K Kawanami D. Association between resistin and fibroblast growth factor 23 in patients with type 2 diabetes mellitus. Sci Rep 2018; 8:13999. 30228288 10.1038/s41598-018-32432-z PMC6143599

